# Partial Cystectomy Management of Bladder Leiomyoma in a Patient With Recurrent Urinary Tract Infections: A Case Study

**DOI:** 10.7759/cureus.56419

**Published:** 2024-03-18

**Authors:** Hussein Abdulwahab Almansour, Akram Bokhari, Abdullah D AlTamimi, Hassan A Alshammari, Yasser H Abd El Moneim Abdo

**Affiliations:** 1 Urology, Hail Health Cluster, Ministry of Health, Hail, SAU; 2 Surgery, University of Hail College of Medicine, Hail, SAU; 3 General Practice, Hail Health Cluster, Ministry of Health, Hail, SAU

**Keywords:** prognosis, minimally invasive surgery, diagnostic approach, female patient, benign tumor, surgical treatment, urologic neoplasm, case report, partial cystectomy, bladder leiomyoma

## Abstract

Bladder leiomyomas are uncommon benign soft tissue neoplasms of the bladder, frequently observed in women. Diagnosis often happens incidentally during ultrasonography, with symptoms varying based on tumour size and location. Here, we present a case of a 57-year-old woman with poorly controlled type 2 diabetes mellitus, successfully treated for bladder leiomyoma through transurethral resection and laparoscopic partial cystectomy. This case highlights the significance of early detection and timely intervention in optimizing patient outcomes for bladder leiomyoma.

## Introduction

Less than 0.5% of bladder tumours are leiomyomas which are uncommon benign mesenchymal neoplasms. The cause is still unclear. Although they might be extravesical, intramural, or submucosal, they originate in the submucosa. Most cases, ranging from 63% to 86%, predominantly manifest in the submucosal (or endovesical) site, while extravesical and intramural leiomyomas represent 11% to 30% and 3% to 7% of instances, respectively [1].

There appears to be a female preponderance among approximately 250 instances recorded worldwide. According to Goluboff et al., women comprised 76% of the cases, with 37 cases reported [1,2]. With an average age at diagnosis of 44 years, bladder leiomyomas appear to be more common in the third and sixth decades of life. A minority of patients describe symptoms such as hematuria and flank discomfort, but the majority 49% and 38%, respectively, present with irritative and obstructive symptoms of the urinary tract [3].

On the other hand, patients could have hematuria, dribbling, hesitation, frequency, pressure of mass effect and urinary blockage [4]. The results of the ultrasound examination are quite characteristic since bladder leiomyomas are smooth, homogenous lesions with peripheral hyperechogenicity [1]. Depending on the location of the lesion, transperineal ultrasound may be a valuable technique for a more accurate assessment. Cystoscopy, CT, MRI, and ultrasound are frequently used in diagnostic procedures [5]. Surgery is the only available treatment option because of the tumour's potential for growth, which depends upon its size, location, and connection to the bladder wall [6]. Similar to uterine fibroids, these tumours are best characterised (site of origin, size, and boundaries) by magnetic resonance imaging (MRI), where they usually show low signal intensity on T2-weighted images and intermediate signal intensity on T1-weighted images [2].

Depending on their size and location, several treatments may be considered for their removal. Transurethral resection is a viable treatment option for tumours that are tiny, submucosal and easily accessible. Conversely, larger, unfavourably positioned leiomyomas may require an abdominal approach for treatment, which reduces the potential of a remaining tumour [2]. Considering its potential benefits, such as lower morbidity, shorter stay in the hospital, and better cosmesis, a minimally invasive alternative to intravesical surgery that may be explored is a laparoscopic technique utilising pneumovesicum. This method has been applied to bladder diverticulectomy, ureteral reimplantation, mesh problems management, bladder foreign body removal, and vesicovaginal fistula treatment [7].

As a very rare cancer, bladder leiomyoma has always been treated with a radical cystectomy, which frequently has a significant effect on the patient's quality of life after the procedure. Partial surgery is now recognised as an effective method to treat a variety of tumours because of its preservation functions and lack of aggression [8].

## Case presentation

**Figure 1:**
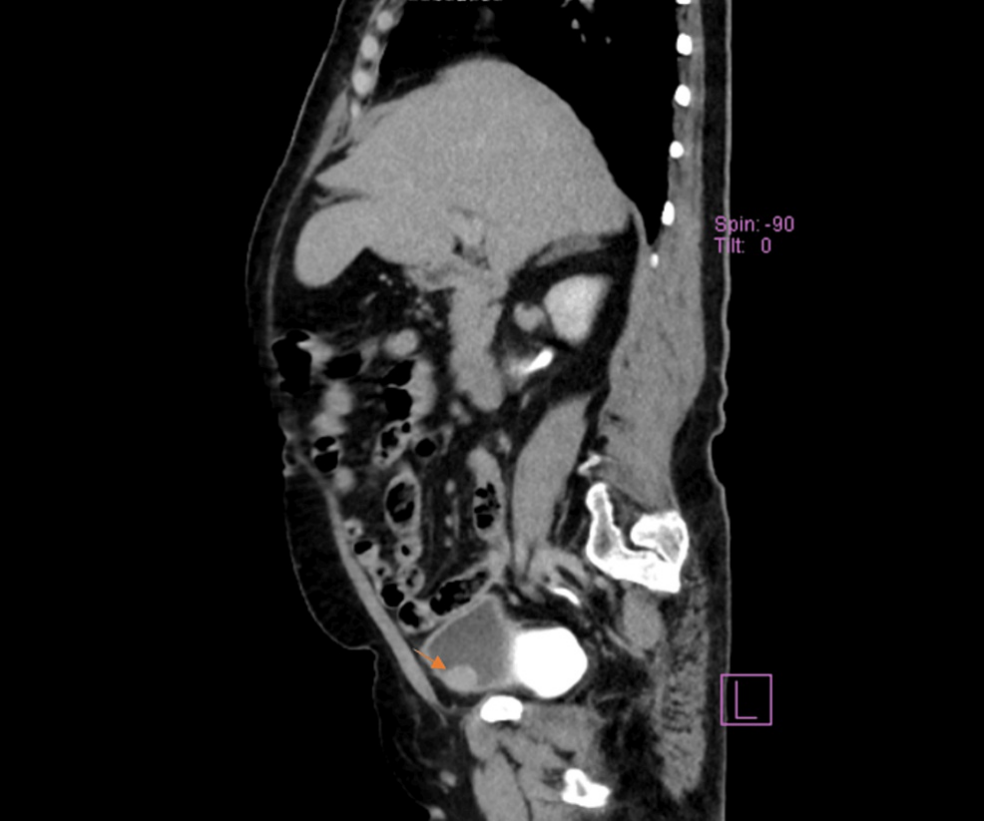
CT abdomen and pelvis without contrast (lateral view) It shows there is a urinary bladder soft tissue lesion seen in the right anterior wall measuring 2 x 1.6 cm in the background of the focal dome wall thickening (arrow). The kidneys are of normal size and show no stones, hydronephrosis, or gross lesions. There is a prominent left inguinal lymph node.

A 57-year-old female with poorly controlled type 2 diabetes mellitus and controlled hypertension, presented to our clinic in June 2023 with complaints of recurrent urinary tract infections persisting for two years. Notably, the patient also has a history of irritable bowel disease. Upon examination, the patient was found to be overweight. No palpable masses or cystocele were noted. Initial investigations including a non-contrast CT scan revealed a soft tissue lesion in the right anterior wall of the urinary bladder, along with normal kidneys and a prominent left inguinal lymph node. Subsequent CT with contrast showed diffuse bladder wall thickening and a focal mass in the right anterolateral wall of the bladder, with reactive lymph nodes (Figure 1). Other organs appeared normal. The impression indicated bladder wall thickening and a focal lesion without intra-abdominal metastasis. The patient underwent transurethral resection of the bladder tumor (TURBT) following consultations with anesthesia, cardiology, gastroenterology, and endocrinology. Notably, the initial non-contrast CT scan was conducted based on the patient's history and complaints.

**Figure 2:**
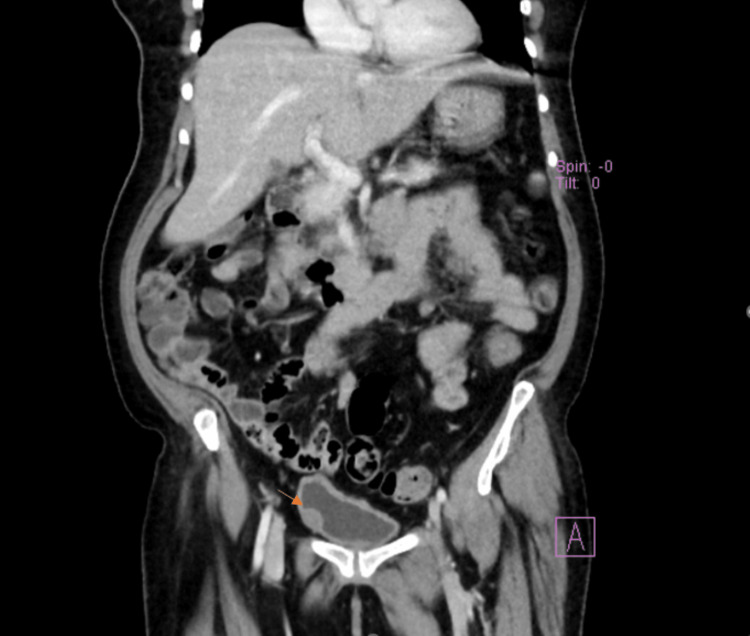
CT abdomen and pelvis with contrast. It shows a dome anterior diffuse urinary bladder wall thickening measuring 0.7 cm and focal right anterolateral wall elliptical shape soft tissue mass measuring 2 x 2 x 1.6 cm (arrow). Both kidneys are normal in size, shape and position with good parenchymal enhancement. There were no renal stones, hydronephrosis or focal lesions. Both ureters have normal lumen diameter, there is no filling defect. The liver, gall bladder, pancreas, and spleen are of normal size and density. No focal lesions. The right and left adrenal glands are bulky.

Subsequent contrast-enhanced CT showed diffuse bladder wall thickening and a 2 x 2 x 1.6 cm soft tissue mass. Bilateral inguinal and left external iliac lymph nodes were identified. Kidneys, ureters, liver, gall bladder, pancreas, spleen, uterus, ovaries, and spine appeared normal in Figure 2.

The patient underwent a transurethral resection of bladder tumor (TURBT), under spinal anaesthesia. Endoscopic resection of a single lesion of the bladder was done.

Under an aseptic measure, a cystoscopy was done. A soft tissue mass under the lateral wall was identified. After that, a resectoscope was introduced, and mass was resected up to muscles. Haemostasis was secured. Using a sterile technique, a fluoroscopically-guided cystogram was performed with Omnipaque contrast infused through the indwelling Foley catheter; 200 ml of contrast was introduced into the bladder through a Foley catheter (as the patient tolerated it).

**Figure 3:**
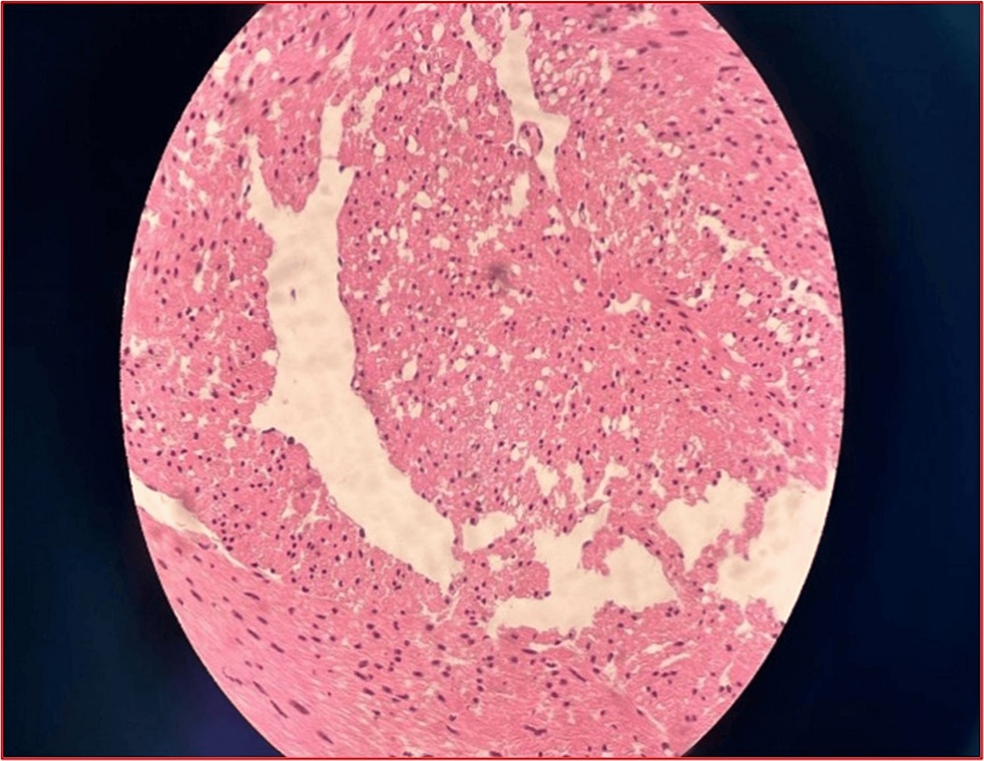
The haematoxylin/eosin stained micrographs showing proliferating smooth muscle fibers disposed in haphazardly arranged fascicles. The component cells show abundant eosinophilic cytoplasm and plump hyperchromatic nuclei in keeping with the smooth muscle.

**Figure 4:**
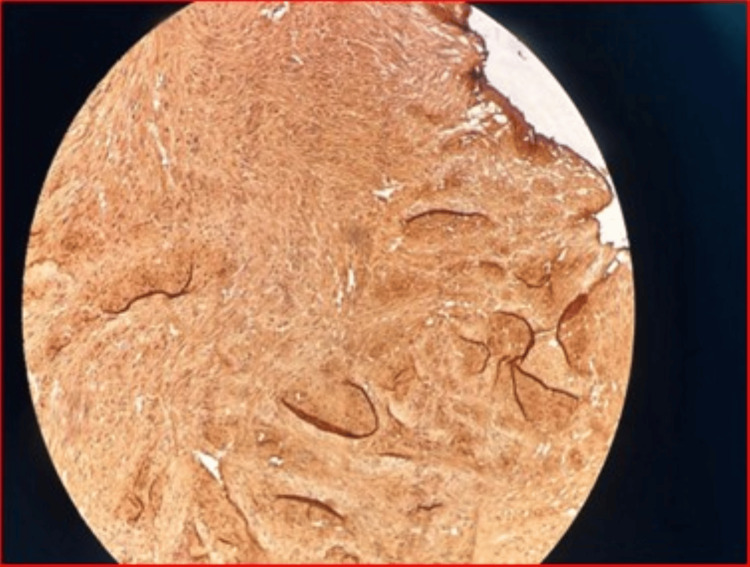
This is a micrograph showing positive cytoplasmic expression of smooth muscle actin (SMA) in proliferation of smooth muscle cells. Positive SMA expression also serves as the histologic diagnosis of a leiomyoma.

Mild irregularities were seen at the superior wall of the urinary bladder. A bladder biopsy revealed fragments of bladder tissue showing benign urothelium overlying a lamina propria with proliferating smooth muscle fibres disposed of in fascicles, shown in Figure 3. There is infiltration of the urothelium and lamina propria by lymphocytes and plasma cells. A focal area of lymphoid aggregates with a germinal centre is seen in Figure 4. There is no focus on dysplasia or malignancy seen in this lesion. Features suggest a bladder leiomyoma with background cystitis, negative for dysplasia or malignancy.

Following the TURBT, the patient underwent a laparoscopic partial cystectomy on November 9, 2023, under general anaesthesia. Healing of the urinary bladder was checked after laparoscopic partial cystectomy. The procedure involved bladder dissection, locating a scar on the right lateral wall, resecting the selected area, and closing the bladder with water-tight testing. No intra or postoperative complications were reported.

## Discussion

Despite the fact that they can occur anywhere in the genitourinary tract, leiomyomas most frequently occur in the renal capsule [9]. The urothelium is where about 95% of primary bladder tumours originate. Mesodermal tumours are responsible for 5% of bladder neoplasms. Over one-third of these cancers are leiomyomas, which account for 0.43% of bladder tumours overall [1], making it the most prevalent benign mesodermal bladder tumour.

Bladder leiomyomas were formerly believed to occur equally in males and females of all age groups [10]. In contrast, Goluboff et al. [2] reviewed 37 patients from medical reports and found that, with a mean age of 44, the majority of the patients were females (76%) in their third through sixth decades (59%). According to the findings of Goluboff et al. [2], all of the patients had an average age of 43.6 years and were female. It is known that bladder leiomyomas can cause symptoms, mostly based on where they are located and how big they are. It is known that bladder leiomyomas can develop endovesically, intramurally, or extravesically, with corresponding frequencies of 63%, 7%, or 30%. The endovesical variety of this group is more likely to produce irritating or obstructive urinary symptoms. According to Goluboff et al., 20% of patients showed no symptoms, and 30% showed severe symptoms associated with hematuria as well as 49% of patients experienced obstructive symptoms [2]. Endovesical leiomyomas have the potential to pedunculate and restrict the bladder's exit [11].

Leiomyomas that grow earlier than puberty are extremely uncommon. These tumours typically arise during the 40 and 50 years of life, when female hormones are secreted at their highest level. Moreover, following menopause, a leiomyoma's size may decrease. Therefore, female hormones may have an impact on leiomyoma formation [12]. Other theories include the fact that women are using ultrasonography at higher rates, which could lead to more female patients being detected. On intravenous urography, leiomyomas can be observed as intravesical filling defects. One sensitive method for diagnosing bladder leiomyoma is ultrasonography. Typically, a plane, uniform, dense mass is seen; nevertheless, reports of leiomyomas that appear partially cystic have been made [13]. T1-weighted and T2-weighted pictures show an intermediate to low signal magnitude, while MRI shows signals with an intermediate magnitude or severity. Following the delivery of contrast material, the tumours will exhibit varying degrees of enhancement; some will show little enhancement, while others will show homogenous enhancement [14].

Even though imaging techniques are beneficial for the process of diagnosis, a histopathologic study is also required in order to verify the diagnosis of leiomyoma and check out the possibility of bladder malignancy. From a gross perspective, the tumours are meaty, whitish, and well-defined. They can be as small as millimetres or as big as 30 cm, weighing anywhere from a few grams to nine kilograms. Uterine and bladder leiomyomas are comparable in terms of microscopical similarities [15].

The accepted standard treatment for bladder leiomyomas is surgical excision. The process of cancer removal is determined by the size, location, and extent of the tumour as well as by the involvement of the ureters or sphincter. Fulguration and TUR can be used to treat small endovesical tumours. Segmental excision is the recommended course of treatment for larger endovesical, intramural, or extravesical tumours. Bladder leiomyoma removal has been effectively accomplished by segmental resection, transvaginal excision, and partial or segmental cystectomy, with minimally invasive surgery known as laparoscopic partial cystectomy and, recently, laparoscopic excision aided by robotics. Certain researchers have proposed that only symptomatic tumours should be surgically removed [16,17].

Because bladder and uterus leiomyomas share histologic characteristics, experience with uterine leiomyomas indicates that asymptomatic patients who have significant chances of a leiomyoma from biopsy and image processing can be maintained without minimally invasive surgery along with cystoscopy analysis. Nevertheless, depending on their location, bladder leiomyomas can only be detected after being surgically removed because they frequently resemble malignant lesions [18]. Additionally, some urologists have experience with partial cystectomies. Therefore, surgical resection is believed to be required for both diagnosis confirmation and effective treatment. Unless urinary tract symptoms arise, follow-up is not necessary because patients with this tumour have a favourable prognosis following surgical treatment, and no malignant change has been observed to date [18].

Although bladder leiomyoma predominates in females, there is a histopathologic similarity to leiomyoma of the uterus and a possible correlation with hormones of females; epidemiological as well as pathophysiologic connection between bladder and uterus leiomyomas remains unclear. This association requires more investigation [1].

It has long been believed that bladder leiomyoma is an extremely aggressive cancer. The majority of patients have been identified in the advanced pathologic stage when the cancer is diagnosed, while less than 15% of malignancies are found in the T1 phase [19]. The disease's metastatic symptoms typically indicate a bleak outlook. When a patient has a disease that has spread locally or internationally, neoadjuvant chemotherapy may be administered. Surgical resection is still the mainstay of treatment regardless of the administration of neoadjuvant chemotherapy, and the state of the surgical margins is a powerful predictor of prognosis [20].

When surgical resection is feasible, the gold standard in these situations is typically an extensive margin radical cystectomy. The quality of life after surgery is greatly reduced by large surgical trauma and the loss of bladder function, even with rigorous attention to standard surgical practice (radical removal of the tumours and complete evacuation of the bladder to surround the uterus, cervix, as well as vaginal cuff in women along with the prostate and seminal vesicles in men) which leads to minimal rates of local tumour recurrence and positive surgical margins [9]. When combined with chemoradiotherapy or laser photocoagulation, transurethral resection of bladder leiomyoma may be performed in certain circumstances (submucosal tiny tumours or individuals who are not able to undergo surgery); nevertheless, this method may not yield better long-term outcomes as compared to surgical excision [8].

## Conclusions

Bladder leiomyoma is a condition that predominantly affects women and is characterized by symptoms influenced by tumor size rather than location. Surgical excision remains the recommended approach for diagnosis confirmation and treatment, with early identification and intervention playing crucial roles in improving outcomes. For smaller tumors, partial cystectomy may offer a preferable option over radical cystectomy, potentially leading to enhanced quality of life and therapeutic effectiveness.
